# Editorial: The molecular mechanisms of epilepsy and potential therapeutics

**DOI:** 10.3389/fnmol.2022.1064121

**Published:** 2022-11-22

**Authors:** Tobias Engel, Gary P. Brennan, Hermona Soreq

**Affiliations:** ^1^Department of Physiology and Medical Physics, Royal College of Surgeons in Ireland, University of Medicine and Health Sciences, Dublin, Ireland; ^2^FutureNeuro, SFI Research Centre for Chronic and Rare Neurological Diseases, RCSI University of Medicine and Health Sciences, Dublin, Ireland; ^3^UCD School of Biomolecular and Biomedical Science, UCD Conway Institute, University College Dublin, Dublin, Ireland; ^4^The Edmond & Lily Safra Center for Brain Sciences, The Hebrew University of Jerusalem, Jerusalem, Israel; ^5^The Alexander Silberman Institute of Life Sciences, The Hebrew University of Jerusalem, Jerusalem, Israel

**Keywords:** epilepsy, epileptogenesis, brain, therapy, anti-seizure medication (ASM)

Epilepsy comprises a heterogeneous group of brain diseases, which all share in common an enduring predisposition to generate seizures. With an incidence of 1–2%, epilepsy is one of the most common chronic brain diseases affecting people of all ages, which in addition to exacting an enormous toll on human potential is also one of the most costly neurological diseases in terms of healthcare and societal costs (Allers et al., 2015). Major challenges in the management of epilepsy include treatment complexity, social disadvantages (e.g., unemployment, stigma), an up to 3-fold increased risk of premature mortality and the presence of numerous co-morbidities such as depression and anxiety (Moshe et al., 2015; Loscher, 2019; Thijs et al., 2019; Shlobin and Sander, 2022). Known causes of epilepsy include genetic abnormalities such as *de novo* mutations and/or a precipitating injury (e.g., traumatic brain injury (TBI), infection, stroke, tumors). In the majority of cases, the underlying causes remain, elusive (Pitkanen and Engel, 2014; Klein et al., 2018). Epileptogenesis, a pathological process transforming a normal healthy brain into an epileptic brain, is characterized by multiple pathological changes within the brain such as acute and ongoing cell death, aberrant synaptic reorganization and neurogenesis, blood-brain barrier (BBB) disruption, and inflammation among many others (Pitkanen et al., 2015). First-line treatment for epilepsy is based on anti-seizure medications (ASM) that are mainly focused on targeting synaptic transmissions and ion channels. Other treatment options include invasive surgery, nerve stimulation and ketogenic diet. Current ASMs in clinical use are, however, only effective in 70% of patients, show no significant impact on disease progression, and may cause serious side effects. Therefore, there remains a pressing need for the identification of treatments with a non-classical mechanism of action, which impact upon disease progression and show efficacy in refractory patients. Pathological changes occurring in the brain during the development of epilepsy remain incompletely understood. In order to design much needed new treatment strategies, we must, however, understand precisely which changes contribute to epileptogenesis and what causes these changes. The goal of this Research Topic was to provide an up-to-date summary of the diverse and complex molecular mechanisms that contribute to epilepsy pathology ranging from inflammatory cells and their role in pathogenesis to the development to post-traumatic epilepsy resulting from TBI.

The outcome volume of this Research Topic comprises eleven articles containing five original research articles and six reviews, including two mini-reviews. The original research article by Schlabitz et al. analyzed changes in gene expression due to epileptiform activity in *in vitro* models of seizures using rodent and human brain slices, which show that *in vitro* seizure models represent a suitable tool to investigate gene expression. Using organotypic hippocampal slice cultures from mice expressing enhanced green fluorescent protein (eGFP) in differentiated granule cells treated with kainic acid, Orcinha et al. show that that the protein Reelin is essential for the maintenance of granular cell lamination in the dentate gyrus and that granule cell dispersion, a pathological hallmark of epilepsy, is the result of a local Reelin deficiency. Using a lithium-pilocarpine status epilepticus rat model, Liu B. et al. investigated the contribution of the gap junction blocker carbenoxolone on dynamic changes in the spectral power of ripples and fast ripples. Their data show that rats pre-treated with carbenoxolone present reduced expression of the gap-junction protein connexin-43, as well as suppressed formation of pathological high-frequency oscillations; thereby providing further evidence of the BBB's impact on epilepsy. Liu R. et al. investigated the effects of imbalanced Na^+^-K^+^-2Cl^−^ (NKCC1) and K^+^-Cl^−^ (KCC2) co-transporters on γ-aminobutyric acidergic (GABAergic) neurotransmission in human focal cortical dysplasia (FCD). The main conclusions of their study is that an imbalanced function of NKCC1 and KCC2 may affect chloride ion homeostasis in neurons and alter GABAergic inhibitory action, thereby contributing to epileptogenesis in FCDs. Finally, Drexel and Sperk investigated whether seizure-induced over-expression of Neuropeptide Y (NPY) contributes to epileptic tolerance by using an animal model based on selective inhibition of GABA release from parvalbumin-containing basket cells in the subiculum/sector CA1. Their results show that NPY overexpression, induced *via* spontaneous recurrent seizures, contributes to epileptic tolerance possibly *via* its actions on the presynaptic Y2 receptor.

In the review article written by Chen et al. the role of interleukin-4 and its impact on glial changes, occurring during epileptogenesis, is discussed as well as its potential as a target for the treatment of epilepsy. Sharma et al. provided a review about mechanisms and risk factors underlying post-traumatic epilepsy with a particular focus on the contribution of neuroinflammatory mediators and immune response factors to the development of epilepsy following TBI and current and novel treatments and management strategies for the prevention of post-traumatic epilepsy. Chen et al. provided a comprehensive review about the latest findings on the possible mechanisms of how N-methyl-D-aspartate receptors contribute to seizures and epilepsy. Mueller et al. provided a general introduction to Designer Receptors Exclusively Activated by Designer Drugs (DREADDs) and how this technique may be applied as treatments for epilepsy. Finally, Wang and Zhao discussed in a mini-review the roles of microRNAs treatments and diagnostic targets for epilepsy and Shaimardanova et al. discussed in another mini-review the potential of gene and cell therapy in epilepsy.

In summary, this Research Topic summarizes and provides new evidence at several different levels relating to its topic and provides useful updates to the readers.

## Author contributions

All authors listed have made a substantial, direct, and intellectual contribution to the work and approved it for publication.

## Conflict of interest

The authors declare that the research was conducted in the absence of any commercial or financial relationships that could be construed as a potential conflict of interest.

## Publisher's note

All claims expressed in this article are solely those of the authors and do not necessarily represent those of their affiliated organizations, or those of the publisher, the editors and the reviewers. Any product that may be evaluated in this article, or claim that may be made by its manufacturer, is not guaranteed or endorsed by the publisher.
